# The Function and Role of the Th17/Treg Cell Balance in Inflammatory Bowel Disease

**DOI:** 10.1155/2020/8813558

**Published:** 2020-12-15

**Authors:** Jun-bin Yan, Min-min Luo, Zhi-yun Chen, Bei-hui He

**Affiliations:** The Second Central Laboratory, Key Laboratory of Integrative Chinese and Western Medicine for the Diagnosis and Treatment of Circulatory Diseases of Zhejiang Province, The First Affiliated Hospital of Zhejiang Chinese Medical University, Hangzhou 310006, China

## Abstract

Inflammatory bowel disease (IBD) is a chronic, inflammatory, and autoimmune disorder. The pathogenesis of IBD is not yet clear. Studies have shown that the imbalance between T helper 17 (Th17) and regulatory T (Treg) cells, which differentiate from CD4^+^ T cells, contributes to IBD. Th17 cells promote tissue inflammation, and Treg cells suppress autoimmunity in IBD. Therefore, Th17/Treg cell balance is crucial. Some regulatory factors affecting the production and maintenance of these cells are also important for the proper regulation of the Th17/Treg balance; these factors include T cell receptor (TCR) signaling, costimulatory signals, cytokine signaling, bile acid metabolites, and the intestinal microbiota. This article focuses on our understanding of the function and role of the balance between Th17/Treg cells in IBD and these regulatory factors and their clinical significance in IBD.

## 1. Introduction

IBD is a disorder that involves chronic and recurrent nonspecific intestinal inflammation with unknown aetiology and pathogenesis. The types of IBD include ulcerative colitis (UC), which can cause long-lasting inflammation and ulcers in the colon, and Crohn's disease (CD), which is characterized by inflammation of the lining of the digestive tract, which can spread into other tissues [1]. IBD can lead to life-threatening complications, including primary sclerosing cholangitis, blood clots, and even colon cancer [2].

The pathogenesis of IBD is still unclear, and the aetiology of IBD may include the host immune system, genetic variability, and environmental factors [3]. In recent years, it has been found that the abnormal intestinal mucosal immune system plays a crucial role in the occurrence, development, and prognosis of IBD, in which the influence of the imbalance in Th17 and Treg cells has been confirmed by previous studies [4, 5]. Multiple factors are involved in the Th17 and Treg cell balance and mainly include TCR signaling, costimulatory signals, cytokine signaling, bile acid metabolites, and the intestinal microbiota.

This paper will discuss the roles of these factors in regulating the balance in Th17/Treg cells and their subsequent influence of IBD, which will provide new perspectives for the treatment of IBD.

## 2. Th17 Cells

As a CD4^+^ T cell subset, Th17 cells play dual roles in the pathogenesis of IBD (mainly a proinflammatory role) [6]. Th17 cells can not only protect the intestinal mucosa by keeping the balance of the immune microenvironment but also exacerbate the intestinal inflammatory response through proinflammatory cytokines. The differentiation process of Th17 cells can be divided into three stages: IL-6 and TGF-*β* initiate the differentiation of Th17 cells, IL-21 expands the differentiation state of Th17 cells, and IL-23 maintains the stable maturation of Th17 cells during the later stage of differentiation [7].

## 3. Treg Cells

Treg cells are a subset of CD4^+^ T cells that play a negative immunomodulatory role and play an essential role in maintaining immune tolerance and balance. Considerable evidence indicates that Treg cells in the intestinal microenvironment contribute to the pathogenesis of IBD [8]. Treg cells mainly participate in various immune diseases by secreting anti-inflammatory cytokines, such as TGF-*β* and IL-10, suppressing the activity of immune cells and thereby controlling inflammation [9]. According to their origin, natural regulatory T (nTreg) cells are primarily generated in the thymus (tTreg) or be generated extrathymically in the periphery (pTreg) in vivo. On the other hand, Tregs generated from naive T cells in vitro, in the presence of transforming growth factor-*β* (TGF-*β*) and IL-2, are called induced Treg (iTreg) cells. Murine studies have shown that tTreg cells exhibit strong lineage fidelity, whereas pTreg cells can revert into conventional CD4+ T cells [10]. Tregs are characterized by forkhead box protein P3 (Foxp3) expression, and Helios is a marker of tTreg for distinguishing tTreg cells from pTreg cells [11].

## 4. Th17/Treg Cell Balance in IBD

Th17 and Treg cells are related through differentiation and in the inhibition of function. They share a common signal pathway mediated by TGF-*β*. Studies have shown that in the presence of IL-6 or IL-21 (with TGF-*β*), naïve CD4^+^ T cells differentiate into Th17 cells; however, in the absence of proinflammatory cytokines, naive CD4^+^ T cells differentiate into Treg cells [12]. Once this balance is broken, a number of autoimmune diseases, including IBD, will occur.

Th17 cells play an important role in the pathogenesis of IBD. Studies have shown that compared with those of healthy controls, Th17 cells infiltrate the intestinal mucosa of IBD patients, and the amount of the cytokine IL-17 that is specifically secreted by Th17 cells increases [13]. In the UC mouse model, Th17 cells in the peripheral blood of mice also increased [14].

Compared with Th17 cells, Treg cells not only suppress the occurrence of autoimmune diseases but also control intestinal inflammation. In the UC mouse model, Treg cells in the peripheral blood of mice decreased [15], and by increasing the secretion of IL-10 and TGF-*β*, the symptoms of diarrhoea in mice were significantly improved [16]. Therefore, Treg cells may be regulated by IL-10, TGF-*β*, and other anti-inflammatory factors that are secreted to suppress the intestinal inflammation cascade and amplify the response, thereby improving the clinical symptoms of IBD. Relevant clinical observations and animal experiments have shown that Treg cells and their inhibitory mechanism are essential for inhibiting spontaneous intestinal inflammation [17]. Therefore, Treg cell deficiency may be the central link in the pathogenesis of IBD.

The regulation of Th17/Treg cell balance is prospective to be a new target for the treatment of IBD. The vitamin A metabolite such as retinoic acid (RA) is the main regulator of the TGF-*β*-dependent immune response, which can prevent IL-6 from inducing proinflammatory Th17 cells and differentiate into anti-inflammatory Treg cells. RA may be helpful in the treatment of IBD [18].

## 5. Factors Affecting the Th17/Treg Cell Balance in IBD

### 5.1. TCR and Costimulatory Signals

The TCR can bind to peptide-major histocompatibility complex (MHC) molecules on the surface of antigen-presenting cells (APCs). In the immune system, the TCR recognizes antigens, transmits signals, and determines the differentiation of T cells [19]. When the TCR-peptide-MHC is on the surface of APCs with the coreceptor CD4 or CD8, it activates the tyrosine-based activation motif (ITAM) at the end of the CD3 chain. Finally, TCR activation enables the differentiation of naive CD4^+^ T cells [20].

TCR signal strength could alter the balance of Th17 and Treg cells [21]. IL-2 inducible T cell kinase (ITK), a critical regulator of intracellular signaling downstream of the TCR, positively regulates the differentiation of Th17 and negatively regulates the differentiation of Treg cells [22]. Studies have shown that attenuated TCR signaling in ITK-/- cells induced immature T lymphocytes to preferentially differentiate into Treg cells but not Th17 cells by inhibiting the Akt/mTOR signaling pathway [23]. Attenuated TCR signals caused by mutations in particular components of the TCR signaling pathway, such as Zap70 [23], DAGs [24], Raf [25], and NF-*κ*B [26], will affect the development of tTreg cells [27]. Studies have shown that attenuated TCR signals did not only promote the growth of tTreg cells but also inhibit [28], such as naïve T cells require weak TCR signals to differentiate into Treg cells [29]. Additionally, aminoacyl tRNA synthetase- (ARS-) interacting multifunctional protein 1 (AIMP1) affects the balance of Th17 and Treg cells directly by downregulating TCR signal complex formation and inducing CD4+ T cells differentiate into Treg cells. In contrast, the differentiation of Th17 cells was not now affected [29]. Bach2, a transcription factor of downstream of TCR signaling, balances TCR signal-induced transcriptional activity of IRF4 to maintain homeostasis of tTreg and pTreg cells and shapes the balance of Th17/Treg cells [30, 31].

The TCR is not sufficient for the complete activation and differentiation of T cells. TCR stimulation in the absence of costimulation will induce anergy or cell apoptosis instead of activation [32]. Therefore, secondary signals are required. Costimulatory signals produced by the interaction of different costimulatory molecules and their ligands could accelerate the differentiation of naïve T cells [33]. CD28 is a costimulatory molecule expressed on the surface of T lymphocytes and plays a crucial role in the activation of T cells. Studies have shown that the costimulatory molecule CD28 participates in the induction of Th17 differentiation [34]. CD28 can also, together with the TCR, upregulate the expression of OX40 [35]. The T cell costimulatory molecule OX40 and its cognate ligand OX40L collectively play an essential role in keeping the growth of Th17 and Treg cells. It was found that the activation of OX40 enhanced Th17 function while blocking OX40L decreased Treg proliferation [36, 37]. However, the effect of costimulatory signals on the balance of Th17 and Treg cells is mainly realized by acting as the second signals of TCR signals.

There are also some coinhibitory receptors, which inhibit the strength of TCR signals. Among them, cytotoxic T lymphocyte antigen 4 (CTLA4) has received much attention. CTLA4, a highly homologous receptor of CD28, competes with CD28 for the same ligands (CD80 and CD86). CTLA4 binds the ligands with a high affinity allowing CTLA4 to inhibit T cell responses by competing with CD28 [38]. Studies have shown that CD28 and CTLA4 have opposing influences on T cell stimulation, and CD28 provides an activating signal, while CTLA4 delivers an inhibitory signal [39]. Costimulatory and coinhibitory signals coassist TCR signals in regulating the balance of Th17 and Treg cells.

### 5.2. Cytokines

Cytokines, small peptides secreted by cells in autocrine and paracrine manners, are the most potent determinants of the fate of T cells. It has been found that the cytokines, which involve in regulating the balance of Th17 cells and Treg cells, are mainly inflammation cytokines, mainly including transforming growth factor­*β* (TGF-*β*), IL-2, IL-6, IL-15, IL-18, IL-2, and IL-23 [40].

TGF-*β* acts on the naive CD4^+^ T cells to induce the development of Th17 cells and Treg cells, while IL-6 induces specific genes in Th17 cells by phosphorylating STAT3, which drives the upregulation of Th17-specific genes, such as ROR*γ*t, Il-17, and IL-23 receptor (IL-23R) [41, 42]. In patients with IBD, TGF-*β* is highly expressed, but TGF-*β*-mediated immunosuppression is significantly impaired. Researchers have found that this effect is related to Smad7, an intracellular protein that binds to the receptors of TGF-*β* and inhibits the Smad-dependent signal transduction driven by TGF-*β*1 [43]. Silencing Smad7 with specific antisense oligodeoxynucleotides can restore TGF-*β*1/Smad signal transduction, downregulate the expression of inflammatory cytokines, and improve experimental UC in mice [44]. The stimulation of naive CD4^+^ T cells with TGF-*β* induces SMAD2 and SMAD3, which in turn activate the transcription factor Foxp3, which could facilitate the differentiation of Treg cells [45, 46]. Unlike IL-6 and TGF-*β*, IL­23 does not directly induce Th17 cell differentiation because of the absence of IL­23R in naive T cells [47]. In mice, T cell receptors engage and bind with specific cytokines, such as TGF-*β*, inducing a network of transcription factors, of which retinoid­related orphan receptor­*γ*t (ROR*γ*t) is the primary regulator and promotes IL­23R expression [48]. IL-23 could thus activate signal transducer and activator of transcription 3 (STAT3) by interacting with IL-23R [49], which promotes the expression of IL­23R and ROR*γ*t, supporting a positive feedback loop that stabilizes the gene expression required for the activation of Th17 cells [50]. IL-21 can also induce the differentiation of Th17 by activating STAT3, which then upregulates the expression of RORrt [51, 52]. IL-18 inhibits the MyD88-dependent downstream signal IL-1R, which in turn reduces the differentiation of Th17 [53]. IL-2 and IL-15 all could upregulate the expression of Foxp3 by activating STAT5, thus promote the differentiation of Treg cells [54]. In addition, IL-15 could inhibit the differentiation of Th17 by reducing the secretion of IL-17 [55]. In addition to inflammation cytokines, HIF-1*α* could promote Th17 differentiation by directly inducing ROR*γ*t transcription as well. HIF-1*α* inhibits Treg differentiation through an active process that targets Foxp3 protein for degradation [42] (Table 1).

### 5.3. Bile Acid Metabolites

Bile acids (BAs) are natural surfactants derived from cholesterol that is produced in the liver and secreted into the duodenum. BAs play a significant role in lipid digestion, antibacterial defense, and glucose metabolism [56]. Through enterohepatic circulation, bacteria convert hundreds of milligrams of bile acid into secondary bile acid with unique chemical structures. Appropriate concentrations of secondary bile acids have immunomodulatory effects [57]. BAs have emerged as modulators of innate immunity and gut inflammation [58].

**Figure 1:**
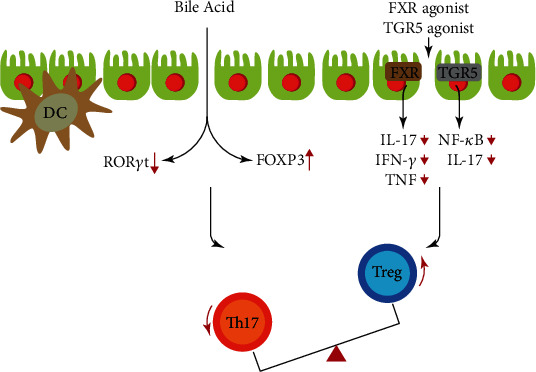
The role of bile acid and bile acid receptor FXR/TGR5 agonist in Th17/Treg balance.

Compared with that of healthy people, the decrease in the abundance of Firmicum in IBD patients leads to a reduction in the level of secondary bile acids, which weakens the anti-inflammatory effect of secondary bile acids and delays the resolution of inflammation [59]. Studies have shown that the expression of bile acid transporter (ASBT) is decreased in TNBS-induced colitis rats and rabbit models of intestinal inflammation [60]. A recent study reported that bile acids control Th17 cell functions by modulating ROR*γ*t activity [61]. Farnesoid-X-receptor (FXR) and transmembrane G protein-coupled receptor 5 (TGR5), the bile acid receptors, regulate innate immunity and the Th17/Treg balance [62, 63]. FXR expression in the inflammatory tissue of Crohn's disease patients was decreased [64]. Clinical observation of using FXR agonist to stimulate IBD patients showed that the secretion of proinflammatory cytokines IL-17, IFN-*γ*, and TNF in lamina propria mononuclear cells was significantly decreased [65]. TGR5 activation of macrophages, which was cocultured with CD4^+^ T cells, inhibited the release of IL-17 in the culture supernatant [66]. FXR and TGR5 agonists may be used to treat IBD (Figure 1).

### 5.4. The Intestinal Microbiota

There are many microorganisms in the gastrointestinal tract that are near related to human health. With the in-depth study of the intestinal microbiota, it has been found that the incidence of IBD is closely related to intestinal microbiota imbalance. Chu et al. [63] noted that intestinal microbiota dysbiosis is the leading cause of immune imbalance and intestinal diseases such as IBD.

Studies have shown that the intestinal microbiota and its metabolites can affect Th17/Treg differentiation. A recent study showed that the microbiotas of humans with IBD could affect the balance of gut Th17 and ROR*γ*t^+^ Treg cells in mice [67]. By using GF mice and antibiotic treatment models, various researchers have found that colonic Th17 cells and Treg cells are significantly decreased in GF mice [68, 69]. Recently, it has been found that ATP and SCFAs, the metabolites derived from the intestinal microbiota, respectively, stimulate the differentiation of Th17 cells and Treg cells [70, 71].

Intestinal microbes and their bacterial products directly act on TLRs and other innate immune receptors to mediate Th17 cell differentiation. A study showed that in the presence of TLR9, intestinal flora DNA can directly induce and promote the differentiation of Th17 cells, inhibit Treg cells, and exacerbate intestinal inflammation [72]. In addition, apoptotic intestinal epithelial cells infected with bacteria also provide ligands for TLRs and activate dendritic cells to secrete IL-6 and TGF-*β*, leading to increased Th17 cell differentiation [73]. The specific role of each TLR in Th17 cell differentiation induction remains to be further explored. One type of filamentous bacteria, *segmented filamentous bacteria* (SFB), induced naive CD4^+^ T cells to differentiate into Th17 cells. Many kinds of research have shown that SFB promotes the differentiation of Th17 cells through serum amyloid A protein (SAA) and dendritic cells (DCs) in intestinal epithelial cells. The cytokine IL-23 secreted by DCs, in turn, can promote an increase in SAA and enhance the secretion of IL-17, thus enhancing the differentiation of Th17 cells and maintaining the intestinal inflammation [74, 75].

Studies on the relationship between the intestinal flora and Treg cells have mostly focused on short-chain fatty acids (SCFAs) and Treg cells. SCFAs can act on intestinal epithelial mucosal cells through TGF-*β*1 to facilitate the differentiation of nTreg cells. SCFAs can also inhibit the activity of histone deacetylase in order to make histones highly acetylated, regulate the expression of related genes, produce anti-inflammatory factors, or lead to growth inhibition and the apoptosis of associated cells, which generates immune tolerance [76]. SCFAs can also affect the ATP level via G protein-coupled receptors (GPCRs) (such as GPR43) or mammalian target of rapamycin (mTOR), thereby influencing the differentiation of Treg cells [77]. In addition, some intestinal flora has been found to alter the Th17/Treg cell balance towards Treg cells by releasing polysaccharide A (PSA); *Bacteroides fragilis* upregulates Foxp3 expression and promotes Treg cell differentiation. Clostridium IV and the XIV flora also induce Foxp3 expression and Treg cell differentiation [78].

**Figure 2:**
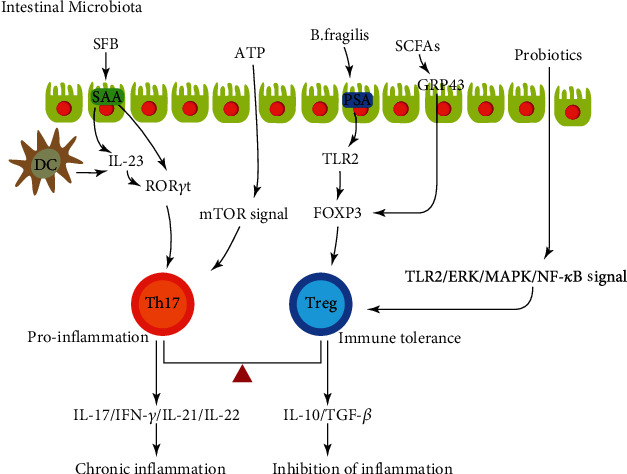
The role of intestinal microbiota in Th17/Treg balance.

A study showed that probiotics could reduce the secretion of TNF-*α* and IL-23 in the serum of mice with oxazolidone-induced colitis. IL-23 is a crucial factor in maintaining the survival, proliferation, and stability of Th17 cells. Therefore, probiotics can inhibit the production and function of IL-17 by reducing the secretion of IL-23, which confirms that probiotics can improve intestinal inflammation in this way [79]. Studies have shown that probiotic *B. adolescentis* can transmit probiotic-mediated adaptive immune regulation to the Treg/Th17 axis through the trl2/ERK/MAPK/NF-*κ*B signaling pathway, stimulate immunosuppressive polarization of macrophages, and secrete the cytokine IL-10 [16]. Probiotics may be used in the treatment of IBD (Figure 2).

## 6. Conclusions

The imbalance of Th17/Treg cells is a vital factor, which influences the occurrence and development of IBD. Th17 cells promote the occurrence of intestinal inflammation and induce autoimmune diseases, while Treg cells inhibit intestinal inflammation. The balance is mainly affected by TCR signaling, costimulatory signals, cytokines, bile acid metabolites, intestinal microbiomes, and other factors. The study of the mutual transformational mechanism of Th17/Treg cells has deepened the understanding of the immune mechanisms of IBD and may provide new research directions of IBD in the future.

## Figures and Tables

**Table 1:** The role of cytokines in Th17/Treg balance.

Cytokines	Mechanism	Changes
TGF-*β*	The stimulation of naïve CD4+ T cells with TGF-*β* induces SMAD2 and SMAD3, which in turn activate the transcription factor Foxp3	Treg↑ [45, 46]
HIF-1*α*	HIF-1*α* promotes Th17 differentiation by directly inducing ROR*γ*t transcription. Also, HIF-1*α* inhibits Treg differentiation through an active process that targets Foxp3 protein for degradation	Th17↑ [42]
IL-2	IL-2 phosphorylates STAT5, which binds to the Foxp3 locus and upregulate the expression of Foxp3	Treg↑ [54]
IL-6	IL-6 drives naïve CD4+ T cells to differentiate into Th17 by phosphorylating STAT3, which then induces the upregulation of Th17-specific genes, such as ROR*γ*t, IL-17, and IL-23	Th17↑ [42]
IL-15	IL-15 upregulates the expression of Foxp3 by activating STAT5 and inhibits the differentiation of Th17 by reducing the secretion of IL-17	Treg↑ [55]
IL-18	IL-18 inhibits the MyD88-dependent downstream signal IL-1R, which in turn reduces the differentiation of Th17	Treg↑ [53]
IL-21	IL-21 induces the differentiation of Th17 by activating STAT3, which then upregulate the expression of ROR*γ*t	Th17↑ [51, 52]
IL-23	IL-23 maintains the differentiation of Th17 by enhancing the transcription of Th17 signature cytokines, such as ROR*γ*t	Th17↑ [50]

## Data Availability

All data generated or analyzed during this study are included in this article.
